# Applying Human-Centered Design to Replicate an Adolescent Sexual and Reproductive Health Intervention: A Case Study of Binti Shupavu in Kenya

**DOI:** 10.9745/GHSP-D-22-00557

**Published:** 2023-12-22

**Authors:** Nancy Njoki, Meghan Cutherell, Abednego Musau, David Mireri, Alex Nana-Sinkam, Mary Phillips

**Affiliations:** a Population Services Kenya, Nairobi, Kenya.; b Population Services International, Washington, DC, USA.; c Population Services International, Nairobi, Kenya.; d IDEO.org, Nairobi, Kenya.

## Abstract

We present a case study describing the use of human-centered design (HCD) to determine how to adapt intervention components from an existing contraceptive uptake program for adolescent girls in 4 geographical contexts (Ethiopia, northern Nigeria, southern Nigeria, and Tanzania) for use in Kenya. First, we prioritized existing intervention components to be tested in Kenya using sacrificial concepts. Through these concepts, we identified key insights and behavioral archetypes from which to build higher-fidelity prototypes, leveraging existing program knowledge and resources while responding to unique opportunities for Kenyan adolescent girls. After 2 rounds of prototyping, we launched a high-fidelity intervention designed to improve contraceptive uptake among girls. We used program experience to identify strategies for improvement during early implementation. The resulting model, Binti Shupavu, is designed to tap into girls' aspirations and connect them with contraceptive use, build their trust in the health system, and work with influencers to build support for adolescent contraceptive use, following the global user journey. In the first year of implementation (January–December 2022), the intervention was scaled from 90 facilities to 360 facilities and reached 60,111 adolescent girls aged 10–19 years. Of these, 21,698 were new voluntary contraceptive users (36%) and an additional 3,873 (19%) were continuing users.

Our design process suggests that HCD is a promising tool for navigating replication. The emphasis on users' perspectives, testing to learn, and collaboration facilitated a deep understanding of the new user population, thus guiding program designers to balance using existing components with developing new ones based on the population's unique needs. Finally, HCD has potential to support the localization agenda if design teams are supported by national, regional, and global experts to be aware of and use the evidence and implementation experience from earlier work.

## Abstract (precis)

The human-centered design process has the potential to serve as a powerful tool for replication of evidence-based interventions, as demonstrated through the case study of Binti Shupavu in Kenya.

## Teaser

**Key Findings:**

- Human-centered design (HCD) has the potential to be a powerful tool for replication of evidence-based interventions and guide decision-making on which program components to adapt and which to maintain.
- An adolescent reproductive health intervention in Kenya provides a case study for the use of HCD during replication to quickly build on lessons from existing contexts while adapting for the new users when needed.
- Binti Shupavu, the intervention resulting from the design process, replicates an existing user journey to support adolescent contraceptive use while still being responsive to the unique needs of adolescent girls in Kenya. In the first year, more than 21,000 girls became new contraceptive users after participating in the intervention.

**Key Implications:**

- Program managers looking to translate evidence-based interventions to new contexts should consider applying HCD processes to effectively build on valuable knowledge and learning while ensuring the intervention meets the needs of the new target audience.
- Public health practitioners working in HCD should share additional experiences of using design for replication to build the evidence base and theory of change for this work.

## INTRODUCTION

In Kenya, 40% of all women of reproductive age are younger than 24 years. Two of 3 sexually active unmarried girls and 1 in 4 married girls aged 15–19 years has an unmet need for contraception.^1^ As girls transition to adulthood, they have a critical need for accurate and relevant sexual and reproductive health information and services. Adolescents, especially adolescent girls, face a wide range of well-documented social, systemic, economic, and political barriers that prevent them from accessing information and support at a time when they are most in need.^2^ These barriers include system-level limitations, such as weak supply chains, laws limiting the provision of contraception to minors, health provider bias, as well as user-level barriers, including social pressure to prove fertility, stigma surrounding use, fear of side effects, and poor knowledge about the available options and correct usage of contraceptive methods.^3^^,^^4^ Designing solutions that overcome these barriers requires innovation and a multisectoral response. There is also growing agreement in the community of practice—researchers, policymakers, and implementers included—that young people have a right to participate in the design and implementation of programs and bring important insights and experiences that can be used to develop more responsive programming.^5^^–^^7^

Human-centered design (HCD) has increasingly been recognized as a valuable tool to tackle the complex challenges that the global health community faces and to address community needs by facilitating faster innovation, better collaboration, and more effective scale.^8^^,^^9^ HCD, as applied to public health, can be defined as an iterative and participatory approach to building global health interventions that achieve health impact in close collaboration with end users.^8^ HCD provides tools that allow teams to quickly and easily test and build on ideas, make strategic pivots based on learning, and make decisions alongside end users in a way that cedes power to participants and gives greater assurance that the resulting intervention will be effective, desirable, and sustainable.^7^ Yet, a potential challenge in the application of HCD in global health is that, to respond to specific end-user needs, the responsiveness of solutions—how “tailored” they are to unique user and context needs—is often prioritized over opportunities to build on already established evidence-based practice.^10^

Transference of evidence-based interventions from one context to another, with rigid adherence to program fidelity and without adapting for new conditions and intended populations, risks less effective interventions.^11^^,^^12^ Guidance for scaling up such practices consistently recognizes the need to adapt interventions to fit with the new implementation context.^13^^,^^14^ Frameworks to clarify the factors affecting implementation of health interventions suggest that characteristics at the structural, organizational, provider, client, and innovation levels influence and interfere with the implementation outcomes of health interventions.^15^ Given the wide-ranging factors of influence, it is not surprising that the evidence base for adapting interventions in new contexts is complex and that the differences across context, team, and health system capacity can make it difficult to understand why interventions succeed or fail in new settings.^16^^–^^18^ Correctly navigating the tension between what program components should be maintained and what should be adapted is one of the most challenging tasks in an adaptation exercise.^16^^–^^19^

Transference of evidence-based interventions from one context to another, with rigid adherence to program fidelity and without adapting for new conditions and intended populations, risks less effective interventions.

Interventions adapted for a new health system must respect the capabilities of that system or risk failure to be sustained. However, interventions with a singular focus on health system capacity, without attention to user needs and preference, especially among vulnerable populations, are likely to be underaccessed and fail to address health disparity.^20^^,^^21^

We present a case study of work done to replicate an intervention targeting improvements in contraceptive use among adolescent girls in low-income countries in a new geography—Kenya. Replication, as we defined it, is the effort to reproduce, in a new setting, the positive results of an intervention that has already been shown to be effective. Rather than copy the existing intervention exactly, we sought to recreate the key components of the intervention in a contextually appropriate way. This allowed us to replicate the essence of the intervention instead of just the form.^19^ We hypothesized that HCD, with its focus on building solutions in partnership with users and rapid iteration based on real-world testing, could provide a structured process to guide decision-making on whether to retain or adapt intervention components to improve fit for the new context. We explain how we used HCD to guide our replication process in 5 counties in Kenya, the resulting intervention, and the lessons learned.

## REPLICATION OBJECTIVE

### Adolescents 360

In 2016, Population Services International and its consortium of partners launched Adolescents 360 (A360) with funding from the Bill & Melinda Gates Foundation and the Children's Investment Fund Foundation. In its first investment phase (2016–2020), A360 worked directly with young people to design and deliver interventions that increase demand for and voluntary uptake of modern contraception among adolescent girls aged 15–19 years. A360 designed and implemented 4 unique interventions across 3 countries—Smart Start in Ethiopia, Kuwa Mjanja in Tanzania, Matasa Matan Arewa in northern Nigeria, and 9ja Girls in southern Nigeria.^22^ Each intervention leads with discussion of girls' aspirations, inclusive of motherhood, and then positions contraception as a tool that can assist girls and, as relevant, couples to achieve their goals. In addition, the approach strengthens the health system to respond to the unique needs of adolescents and to provide adolescent girls with a full array of short- and long-acting contraceptive method options in a supportive environment. The external evaluation of A360's interventions showed their promise in addressing the reproductive health needs of adolescents. At a population level, although the interventions did not lead to increased use of modern contraceptives across all geographies, they demonstrated improved outcomes among girls who were exposed to the interventions. The evaluation concluded that these novel approaches needed to be accompanied by intensified efforts to address the contextual drivers of low modern contraceptive use, especially when intended for unmarried adolescents who face unique stigma around contraceptive use.^23^^,^^24^

**FIGURE 1:**
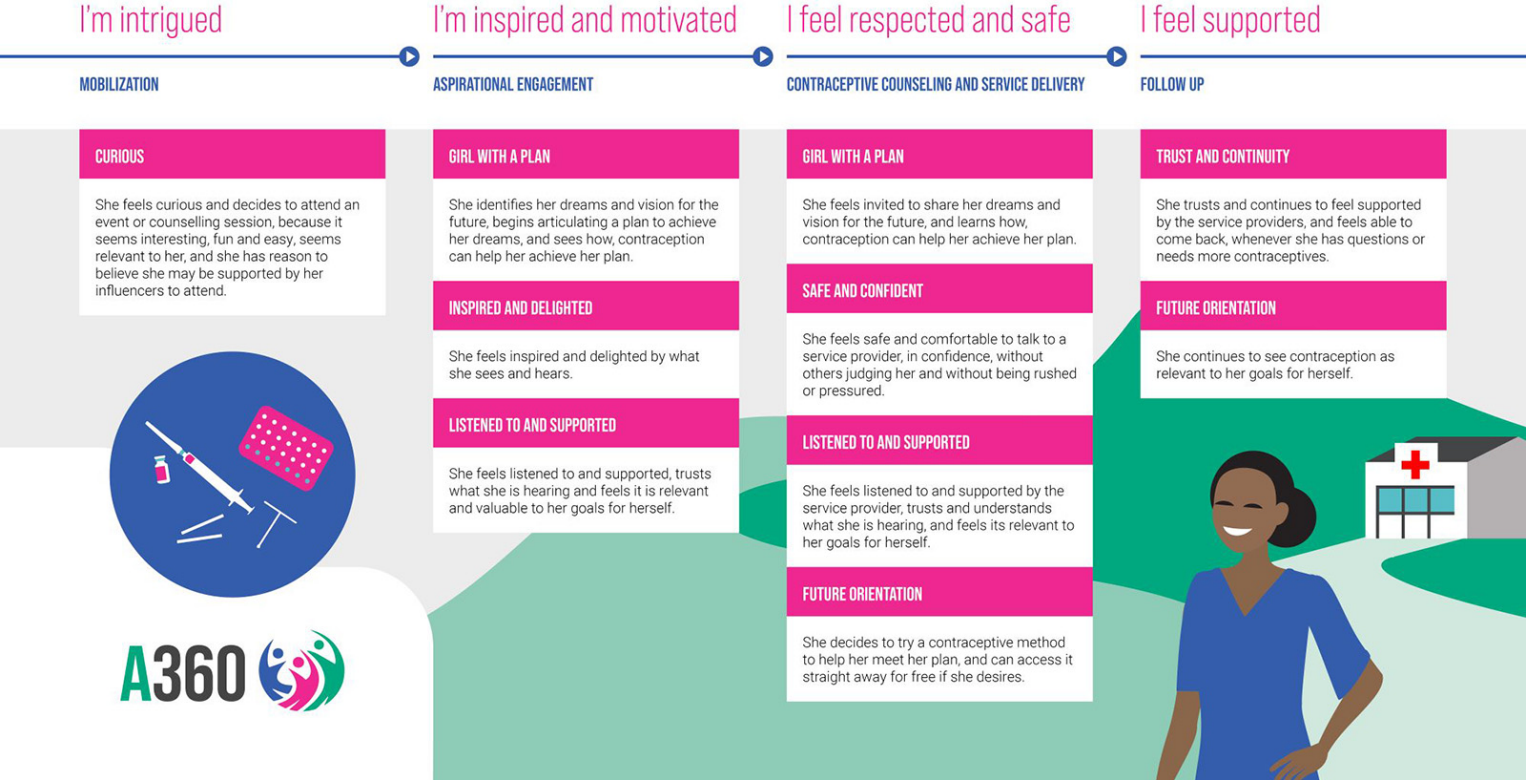
Summary of the A360 Global User Journey

The A360 Global User Journey is a visual representation of key touchpoints the programming offers adolescent girls to support their decision to take up and continue to use a contraceptive method and reflects the general activities (mobilization, aspirational engagement, counseling and service delivery, and follow-up), as well as the mindsets and emotions, the interventions seek to prompt at each stage (Figure 1).^25^ This journey is a synthesis of our experience in Ethiopia, Nigeria, and Tanzania and served as a blueprint for replication activities.

### Replication in Kenya

In its second investment phase (2020–2025), A360 received funding to expand its programming into Kenya. With HCD as a key tool, the project crafted a strategy to replicate its global user journey in Kenya, building on components of the previously designed interventions as well as lessons learned from the first phase of programming to accelerate the timeline. Additionally, we sought to use this replication in Kenya as an opportunity to strengthen areas identified for improvement in the first investment phase. Priority areas included long-term potential for institutionalization in government systems for sustainability and more emphasis on gender-transformative programming addressing contextual barriers to contraceptive use. This process aimed to generate a final intervention design in Kenya that built on our existing user journey for contraceptive uptake and continuation among adolescent girls while also ensuring the output was uniquely suited to the needs and preferences of adolescent girls in Kenya.

## REPLICATION APPROACH USING HCD

The design team consisted of program staff from Population Services Kenya, A360's local implementing partner in Kenya, technical support from Population Services International staff based in the United States and Kenya, and members of IDEO.org who were based in Kenya and the United States. Due to COVID-19 restrictions, only members of Population Services Kenya participated in program implementation work. For this process, we adopted the IDEO.org HCD framework because of its simple structure, focus on design mindsets, and the design team's familiarity with it from previous HCD work.^26^ On top of this framework, we layered subphases to create clarity on the goals and steps of each part of the process, taking advantage of the existing body of evidence and experience that was generated in the first phase of A360. The process outlined in the framework is the same process we followed to develop the interventions in the program geographies in the first phase. Two notable additions include the decision to use design briefs to define our objectives and to begin design implementation work with sacrificial concepts rather than from scratch.

Sacrificial concepts are early, raw concepts that are made visual or physical and used as a tool to encourage reactions, responses, and discussion among users and design teams.^27^ They are designed to be quickly discarded if they do not work well. Typically, when used early in a design process, these are highly conceptual and may be provocative or outrageous, designed to elicit substantial feedback and insight. We chose to use stripped-down components of our own programming as sacrificial concepts, hypothesizing that there would be 2 benefits to this approach. First, we knew why different components worked in our other geographies; if something worked or did not work in Kenya, we might be able to assume a similar underlying condition or motivation. Second, these components were all well developed, with assets, facilitation guides, and multiple iterations. If a rough concept was appealing in Kenya, it would be easier to develop the prototype to a higher fidelity.

**FIGURE 2:**
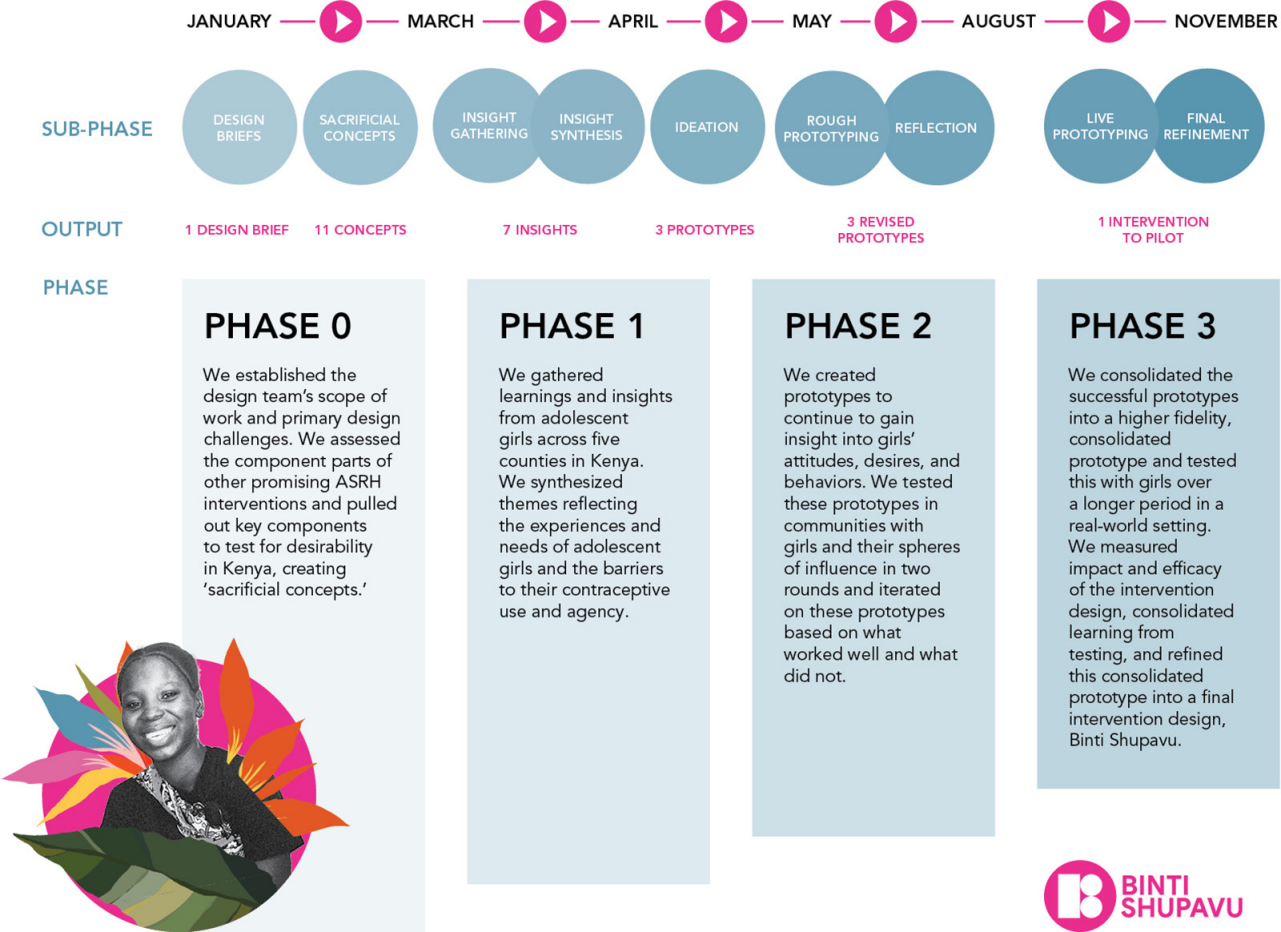
Replication Intervention Design Approach Timeline Phases Abbreviation: ASRH, adolescent sexual and reproductive health.

To support the emphasis on replication, our design partner introduced and encouraged the mindset of “designing forward,” with a focus on creating low-fidelity prototypes to test existing assumptions, thereby accelerating the insight-gathering process. These subphases were mapped to the project timeline (Figure 2).

## METHODS

### Study Setting

We conducted the HCD process in 5 counties across 10 subcounties: Kabondo Kasipul and Karachuonyo (Homabay), Suna East and Suna West (Migori), Kajiado Central and Kajiado East (Kajiado), Narok North and Narok South (Narok), and Kilifi South and Magarini (Kilifi). Given their demographic similarities and geographic proximity, we grouped Homabay and Migori together in a research and design cluster and Narok and Kajiado together while Kilifi remained on its own. As part of a process evaluation, the project's learning and evaluation partner conducted qualitative research on the replication activities and collected lessons from the perspective of designers and programmers based primarily in Kenya and the United States.

### Participant Recruitment

Participants for the design and process evaluation were recruited using purposive sampling to ensure diversity of respondents within the subpopulation of adolescent girls (married, unmarried, in-school, out-of-school, and with and without children). Community health volunteers, health providers, and the program team members familiar with the community used recruitment scripts to identify and invite potential participants. Recruiters linked eligible and interested individuals to the design and research team. All participants received detailed information about the design and evaluation process, its goals, and expectations for their involvement and provided written consent before their involvement.

### Data Collection Methods

During the design process (January to November 2021), the design team engaged 677 adolescent girls, 49 husbands of adolescent girls, 195 mothers of adolescent girls, and 145 community influencers (including local government officials, religious leaders, chamas [savings group members], and leaders of community-based organizations). In each phase, we included adolescent girls and their key influencers. We applied a range of methods drawn from qualitative research and design practice with the goal of deepening our understanding of our audience and how best to address their barriers to contraception use (Table 1). For all data collection methods, the tools were developed in English and translated into the local language in each county: Dholuo in Homabay and Migori, Masai in Kajiado and Narok, and Kiswahili in Kilifi. The design team responsible for data collection consisted of trained youth innovation champions (Kenyans aged 18–25 years employed by the project to build rapport and trust with interview participants, as well as contribute their lived experience as young people), research assistants, and county health officials. The design team used an iterative process, choosing from a predefined set of data collection tools but maintaining flexibility to shift between different methods in response to the insights gathered in real-time from participants. This iterative process allowed teams to be more experimental and quickly discard prototypes that did not work well. In turn, it allowed the team to focus on methods that provided deeper insights and fostered meaningful engagement between the design team and participants.

**TABLE 1.** Design Methods Used by Design Phase and Type of Participants Involved to Replicate an Adolescent Sexual and Reproductive Health Intervention in Kenya

HCD Phase	Methods	Participants
Insight gathering and synthesis	Individual interviews Focus group discussions Photo-narratives: Participants are asked to spend a full day taking photos of their daily activities and describe these to data collectors.	29 adolescent girls 9 community influencers
Prototyping	Skits: Data collectors either act out or ask participants to act out a fictional scenario to gain audience reactions. Card sorting: Data collectors provide participants with a set of cards on a specific topic and ask them to sort them in various ways and provide reasons for their responses. Vox props: Data collectors provide a short prompt to participants (often in the form of a story) and ask them questions to understand their reactions and opinions. Sample advertising: Data collectors present different branded materials and ask participants to explain what they do and do not find appealing.	78 adolescent girls 16 husbands of adolescent girls 21 mothers of adolescent girls 34 community influencers
Live prototyping	Small-scale implementation: Design teams create a version of the intervention that can be run under real-world conditions to assess feasibility. Data collection methods include participant observation, quantitative monitoring of behaviors, and qualitative interviews to understand what appealed to participants and what did or did not prompt new behaviors or outcomes.	570 adolescent girls 33 husbands of adolescent girls 174 mothers of adolescent girls 102 community influencers

Process evaluation activities took place between May and December 2021 and included 2 focus group discussions with adolescent girls in Kajiado (n=10) and Kilifi (n=8) counties and 14 in-depth interviews (8 Kenya-based program members, 3 health workers in Kajiado and Kilifi counties, 2 global staff members, and 1 staff member from IDEO.org). Process evaluators also participated in key reflection moments in the design process and reviewed project documents.

Program monitoring data were used to track implementation following the design phase. Providers located at participating health facilities captured key performance indicators (Table 2) using existing contraceptive uptake indicators in the national health information management system that were reported and visualized on DHIS2. For indicators not available through the health information management system, we implemented a parallel data collection system consisting of intervention activity tools.

**TABLE 2.** Key Performance Indicators Captured to Monitor Adolescent Sexual and Reproductive Health Intervention Performance Post-Design in Kenya

Indicator	Definition	Data Source
Girls reached	The number of girls aged 10–19 years who attend Binti Shupavu activities.	Intervention activity tools
New contraceptive users	The number of girls aged 10–19 years who are new to the facility and take up a modern contraceptive method after a Binti Shupavu event. Among new users, data are disaggregated by age and by method.	Health management information system
Continuing contraceptive users	The number of girls aged 10–19 years who have already received a method from this facility and who receive a refill/reinsertion or a new method after a Binti Shupavu event.	Health management information system
Conversion rate	The proportion of girls attending Binti Shupavu activities adopting a modern method after a Binti Shupavu event, less the continuing contraceptive users.	Intervention activity tools

### Data Analysis

Notes and interview responses were initially written by hand by design team members. Additionally, some interviews were recorded, and an assigned research assistant managed the digital files and was responsible for transcription after the end of the implementation work. Each day, the team gathered for “downloads,” where team members shared participant responses that were notable or surprising, including quotes. As a result of travel limitations due to COVID-19, we leveraged a digital whiteboard platform (Miro) to record and document the process with the global design team. Data were recorded directly onto Miro or written in a Word document and later transferred to Miro. After the completion of data collection, the design team met to review the data. Data were first grouped deductively into key themes. These themes were then reviewed by a multistakeholder team and used to develop insights that spoke to realities or challenges on the ground or key areas of interest for the design process. These insights were reviewed by a team of technical experts for accuracy, usefulness, and originality before being finalized. Transcripts from the process evaluation were analyzed by the research team deductively using thematic analysis as suggested by Braun and Clarke.^28^

### Ethical Approval

Ethical approvals were obtained for all phases of the design process as well as for process evaluation activities from the AMREF Ethics and Scientific Review Committee (approval number P926-2021) in Kenya. Permissions were obtained from the relevant government departments from the 5 counties.

## HCD PHASES

### Insight Gathering and Synthesis

We began the insight-gathering and synthesis phase with the goal of rapidly understanding similarities and differences in Kenya compared to the earlier project geographies. With this understanding, the design team could make more informed decisions about which intervention components were likely to be effective. To do this, the team began by studying the interventions developed for other geographies in the first phase, breaking them into their component parts and creating rough prototypes of those components to serve as sacrificial concepts. To complement this, we also used traditional inquiry methods, such as in-depth interviews and focus group discussions with the participants listed in Table 1.

**FIGURE 3:**
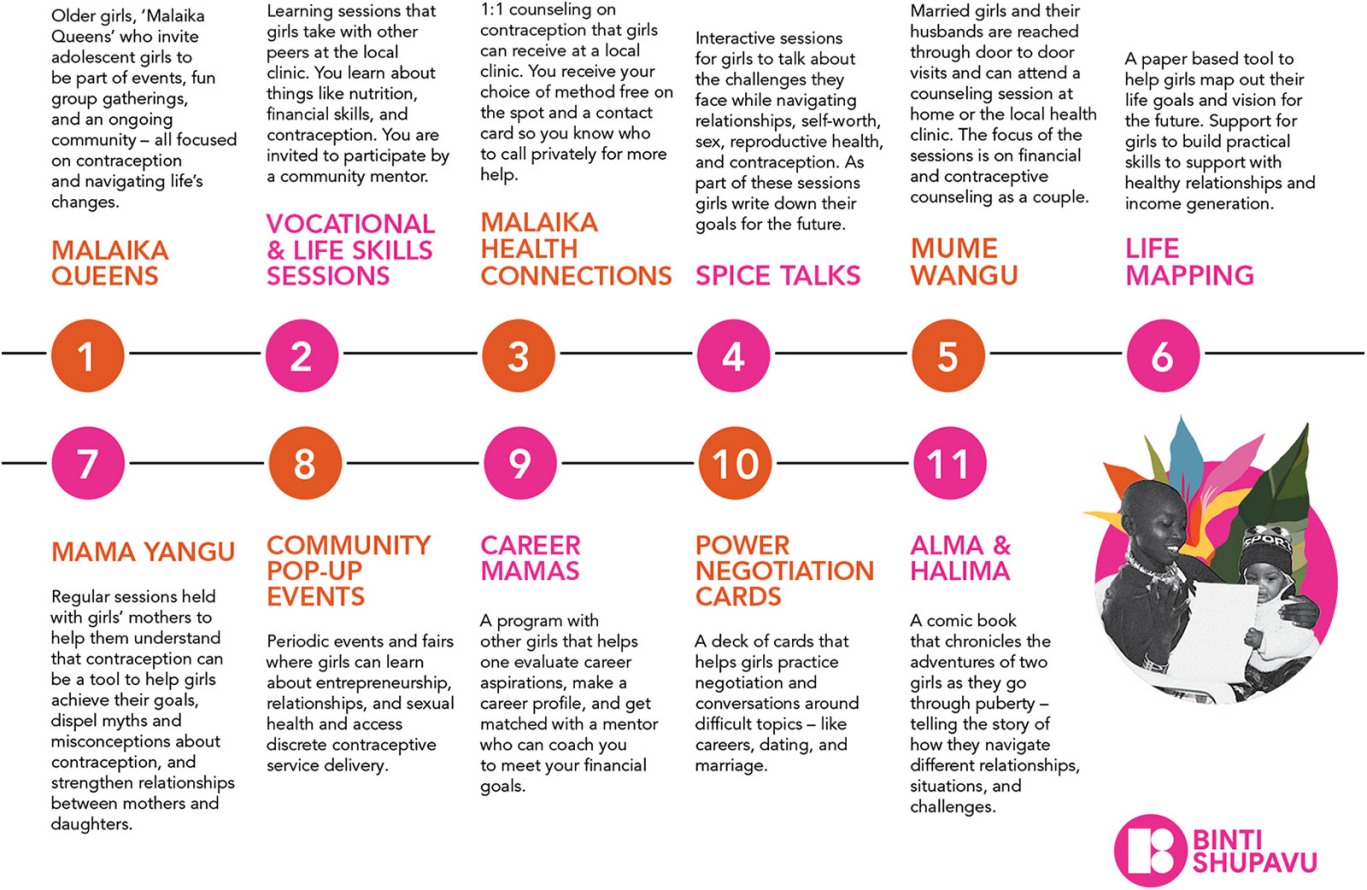
Sacrificial Concepts to Determine Topics of Interest for Adolescent Sexual and Reproductive Health Intervention

Together, the design team assessed the different interventions and prioritized the 2 that were the most promising for the selected counties in Kenya based on shared demographics and cultural context. We chose the interventions with unmarried adolescent girls in southern Nigeria (9ja Girls) and Tanzania (Kuwa Mjanja). Additionally, the team examined the components of each intervention and chose those considered core to driving voluntary contraceptive uptake and continuation while in need. This process resulted in a final set of 11 sacrificial concepts (Figure 3).

The design team assessed the different interventions and prioritized the 2 that were the most promising for the selected counties in Kenya based on shared demographics and cultural context.

Girls' and influencers' reactions to the sacrificial concepts, as well as the corresponding interviews, helped us understand the topics that interested girls, how they thought about and accessed health services, and the intervention components that were attractive to this audience. Using these data, the team synthesized 7 key insights. These insights provided an initial idea of the design opportunities that existed to address the barriers girls encountered to contraceptive use and autonomy (Table 3). The insights were consistent with the barriers identified in the global evidence base and similar to those identified in other project geographies. This validated for the design team that it made sense to try and replicate the existing user journey. Design opportunities were articulated in the form of “how might we” questions, which reframe the problem to encourage the generation of solutions.

**TABLE 3.** Themes From Insight Synthesis and Design Opportunities to Address Barriers to Adolescent Girls' Contraceptive Use and Autonomy

Insight	Description	Illustrative Quotes	Design Opportunities
Wayfinding amid an ocean of misinformation	Despite girls' curiosity to better understand contraceptive options, accessing information often felt overwhelming and confusing without clear support in navigating misinformation.	“If the methods don't work with your blood… you can easily get pregnant.” —Adolescent girl in Migori “When I hear contraception… it means the user doesn't want to get many children.” —Adolescent girl, Kajiado	How might we design approachable and convenient access points for girls and their surrounding communities to receive accurate information? How might we frame education around myths and misconceptions as a community-wide goal and not just a burden on girls?
Experiencing fear at the forefront	Girls experienced fear, shame, and uncertainty when learning about contraceptive methods and thus did not feel empowered to explore their bodies or their choices.	“When you adopt a contraception method you must hide it so that your parents & friends won't consider you as a person with loose morals.” —Adolescent girl, Narok	How might we equip girls to identify, express, and overcome the fears that they are experiencing when learning about contraceptive methods? How might we support the community surrounding girls to understand the impact they have on how she feels about her body and choices throughout adolescence?
Discretion and privacy	Girls valued access points to discuss their concerns and options but required more discretion than was often offered to feel safe in expressing themselves openly.	“I tell or ask my family or a friend because then it remains private.” —Adolescent girl, Homabay	How might we create opportunities for girls to discreetly engage in conversations about SRH on their own terms? How might we design SRH programming that offers girls both the support of an encouraging community alongside the right amount of discretion and privacy?
Enabling the community to support and champion girls	The community surrounding girls significantly impacted her beliefs and concerns; although most mothers, fathers, and partners wanted to help, they lacked the information and exposure needed to guide the adolescent girl on her SRH journey.	“Boyfriends & men don't know much & also don't want to hear that you have adopted contraceptives because they also have similar concerns about its negative effects.” —Adolescent girl, Homabay “Nani kama Mama –who is better than mum” – she won't fear to tell you the truth and she wants the best for you in life.” —Adolescent girl, Kilifi	How might we enable role models and influencers to support girls in accessing supportive information when making SRH-related decisions? How might we build awareness with role models and influencers as to the importance of their role and the potential of their influence on a girl's life and decision-making power?
Her experience in facilities, her relationship to providers	Health facilities were where girls needed to go to get access to information and services, but they didn't trust providers, nor did they feel like these spaces were for them.	“I was in the hospital and the doctor told me that these methods are being phased out because of the side effects. Especially the injection method.” —Adolescent girl, Migori	How might we engage and equip health facilities and providers to strengthen their youth and girl-friendly services and offers? How might we support girls in understanding their rights, building a practice of open expression of concerns and shameless desire for informed decision-making about their bodies?
Building her confidence for the future.	Adolescent girls' dreams were bound by their exposure to what was possible. Girls were well aware of the difficulties and challenges of building toward nontraditional goals, like continuing education or entrepreneurship. She needed a “reality check” but also belief from those surrounding her that she was capable, despite the challenges, and that she could learn and grow toward more.	“We are a digital generation and you should consider upskilling us with sophisticated skills -not the usual basics.” —Adolescent girl, Migori “The boy child should also be included in the education, so that they [support us and] don't idle around and get us into temptation.” —Adolescent girl, Homabay	How might we ensure programming includes resources and tools that equip the adolescent girl to dream forward for herself – whether that's starting a family, continuing her education, or becoming a businesswoman? How might we consider dissolving the gender barrier in future-forward programming, including the perspective of the adolescent boy and seeking to transform adolescent boys into supporters, collaborators, and advocates?
Self-expression, optimism, and ownership	She was excited and curious about her future; she craved support to build her confidence and take ownership over her own story.	“Girls would like to have the ability to make important choices that affect their lives and would like to be empowered enough to avoid overdependence on their communities, who could impose their decisions on them.” —Service provider, Kilifi “Having girls themselves take on a visible leadership role within the community and a sense of responsibility can be positive forces for change in the lives of young girls.” —Community influencer, Kilifi	How might we create an environment in which girls are confident to take up space, speak up, claim ownership of their decision-making and feel fully engaged in intervention activities? How might we prioritize components that are cocreated with girls, integrating their perspectives and preferences into the final product?

Abbreviation: SRH, sexual and reproductive health.

### User Archetype Identification

**FIGURE 4:**
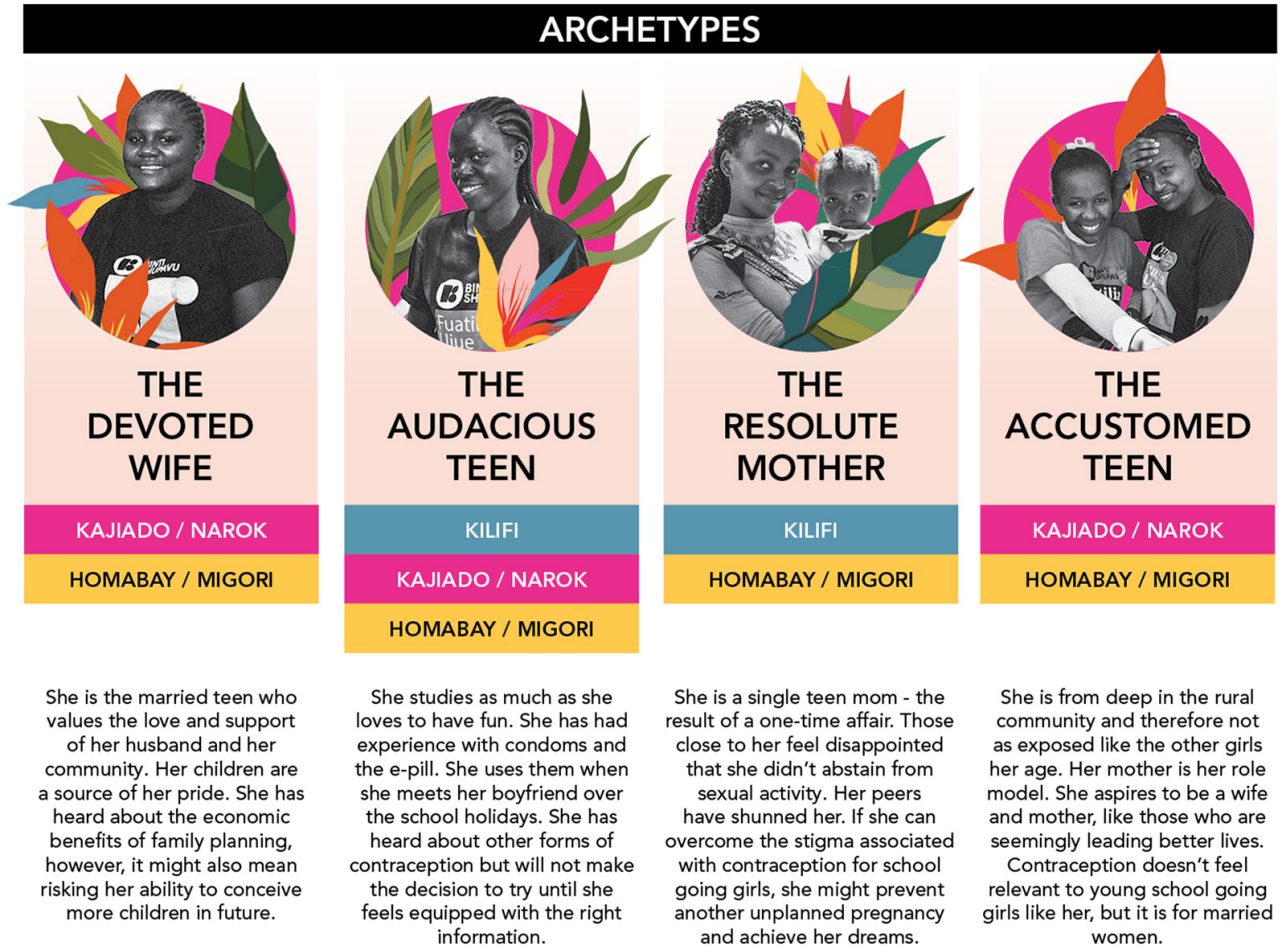
Adolescent Girl Behavioral Archetypes Used to Develop an Adolescent Sexual and Reproductive Health Intervention in Kenya

An additional activity during this phase was to develop behavioral archetypes of the different adolescent girls based on findings from insight gathering (Figure 4). Archetypes (sometimes known as personas) are a way to synthesize insights collected to define the needs, goals, and challenges of different types of user groups.^29^ We used the archetypes to create clarity on which type of girls the intervention should be designed for, as the archetypes showed that subpopulations of girls had different levels of need for and barriers to using contraception. With limited resources, the project had to narrow its focus to priority archetypes to develop successful solutions. Across the archetypes, all girls demonstrated a strong desire to access information about sexual reproductive health to make their own choices about their bodies and their futures. The team chose to focus on the “devoted wife” and “resolute mother,” as these 2 groups offered the greatest potential for impact (unmet need for contraception) and scale (largest population). The use of behavioral archetypes in addition to demographic data allowed the design team to deepen its understanding of which existing interventions were most promising in the Kenyan context. The “resolute mother” was similar to the girls reached in Tanzania, but the additional focus in Kenya on the “devoted wife” meant that the design team could consider components from our programming for married girls in Ethiopia and northern Nigeria. These decisions also meant that key influencers we needed to reach would include both mothers of the adolescent girls (who were often still helping to support the “resolute mother” group) and husbands.

### Prototyping

**FIGURE 5:**
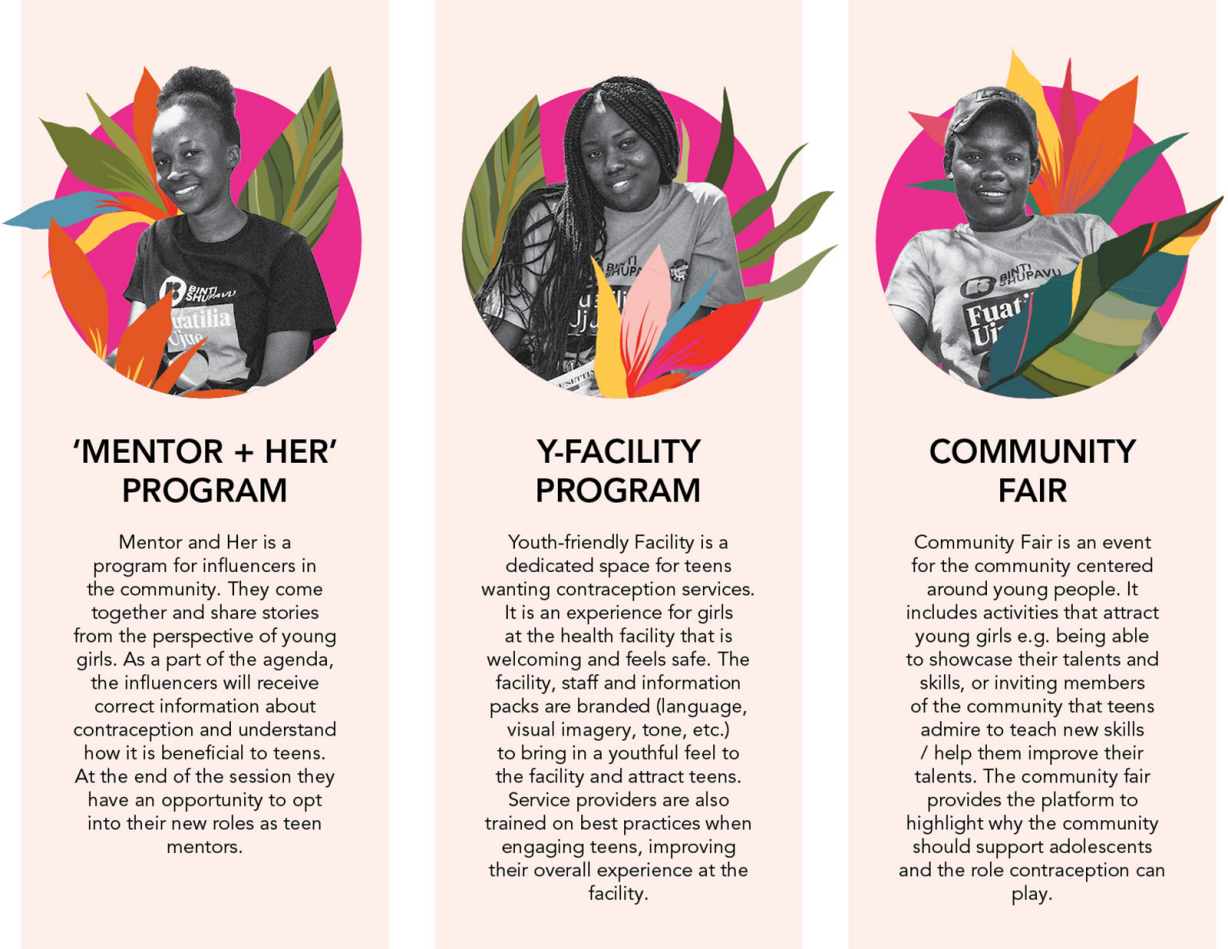
Prototype Concepts for Adolescent Sexual and Reproductive Health Intervention

The design team then used the insights, opportunities, and archetypes to build a set of prototypes (Figure 5). A prototype is a preliminary version of a product or service that invites the end user to experience the design idea and is realistic enough for the user to provide substantive feedback but does not require substantial resources to build. Findings from this phase helped our design team test in small-scale ways intervention components that would be effective among Kenyan adolescents and their influencers in attaining better SRH outcomes.

These prototypes built on the initial sacrificial concepts identified as promising after the insight gathering phase. As concepts were developed, we incorporated additional components from the project's other interventions to make them more robust. For example, because mothers were identified by girls as key influencers, the project was able to adapt messages developed in southern Nigeria for mothers of adolescent girls. At the same time, there were some unique prototypes proposed in Kenya because of specific needs and opportunities identified in this context, as well as our added focus on programming for the enabling environment. Although the team borrowed the messages from Nigeria, they found that audiences in Kenya responded better to storytelling than lectures or information-sharing, and so developed ways to share key messages with mothers in narrative form from lived experiences. In the project's first phase, early-stage testing moved slowly, as we were uncertain about the pathway to contraceptive uptake for adolescent audiences. In the replication process, we were able to use the established user journey as a guide and focus on replicating the specific activities, mindsets, and emotions for each step. We were able to draw from many well-developed scripts and delivery approaches, which allowed us to build more complex prototypes faster and to be alert to what was new or similar from previous experiences, permitting us to learn faster. This process reflects a distinctive value in the replication design process, which allowed for building on earlier lessons while underlining the importance of leaving space to create something new for a unique context (Table 4).

**TABLE 4.** Kenya Prototype Replication Influences From A360's Other Interventions

Kenya Prototype	Replication Influences	Unique Kenya Components
Mentor + Her/Mastory za Kikwetu	9ja Girl's (southern Nigeria) approach to influencer engagement, particularly the messages used in mothers' sessions Kuwa Mjanja's (Tanzania) parents' sessions designed to build empathy for adolescent girls	Storytelling to build empathy and spark discussion around girls' agency and the place of contraception. Stories were taken from adolescent girl testimonials and recorded on video or audio.
Y-Facility	Kuwa Mjanja's existing in-clinic service model Service delivery model in both 9ja Girls and MMA (northern Nigeria) 9ja Girls' “life mapping” activity	Dedicated adolescent-girl service days at government-run health facilities to support the goal of sustainability and institutionalization into government systems.
Community Fair	Kuwa Mjanja's out-of-clinic models, particularly the opportunity for adolescent girls to see skills demonstrations and understand the opportunities available to them in their communities. Curriculum from 9ja Girls' skills classes (goal-setting, budgeting and saving, health and nutrition, decision-making and communication, and income-generating skills sessions.)	Instead of being an event dedicated only to girls, this was a moment to pique girls' and community members' interest about the intervention and for girls to showcase their skills to others and mobilize additional girls into the intervention.

**FIGURE 6:**
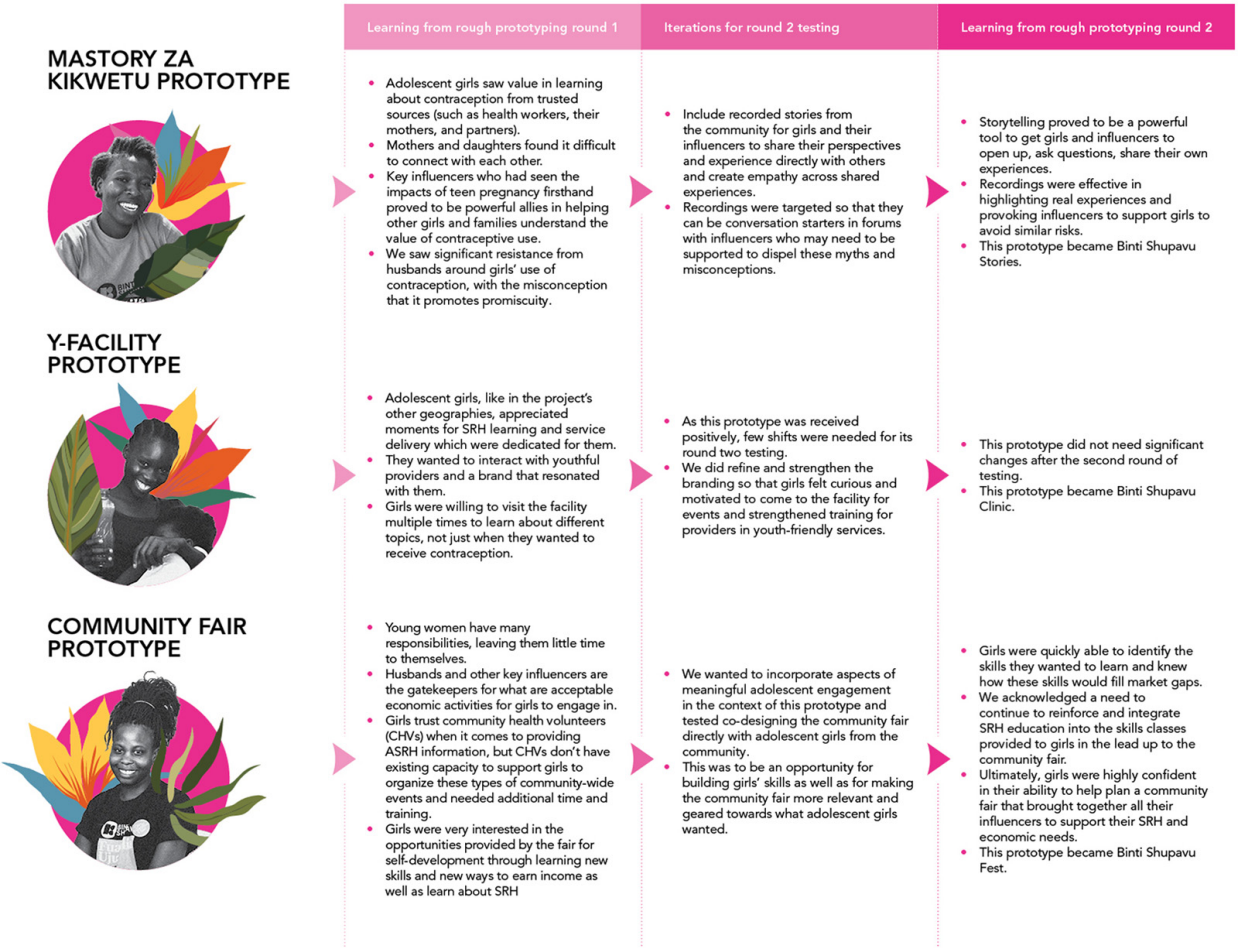
Binti Shupavu Prototypes With Iterations Incorporating Feedback Abbreviations: ASRH, adolescent sexual and reproductive health; SRH, sexual and reproductive health.

We tested these prototypes in 2 rounds. During testing, adolescent girls and their influencers were asked whether they would like this type of intervention, how they might react when presented with the intervention, and other questions to gauge desirability of the prototypes. In between rounds of testing, the design team synthesized these responses and made small, iterative changes to incorporate feedback to enhance desirability for girls and their influencers (Figure 6).

### Live Prototyping

At the end of the prototyping phase, the design team consolidated the 3 concepts, each of which had a specific role in the user journey, into a holistic intervention system. We ensured there were clear linkages between the different prototypes, for example, between Mastory Za Kikwetu (formerly Mentor + Her) and Y-Facility sessions, to ensure girls understood the value of engaging with different intervention components and would be referred for different types of services across the intervention, depending on their unique needs.

Before live prototyping, we also partnered with girls through a cocreation process to generate a new name and cohesive branding for the intervention. Branding is an important component of effective adolescent sexual and reproductive health interventions. We have found that a country-specific or culturally specific brand can help build trust and credibility with the intervention and help adolescent girls see that the product is intended for them. Brands help build visibility of the intervention, build cohesion between components, and create ownership among girls, in contrast with traditional contraceptive delivery interventions that are typically branded without user or cultural consultation. Together, we decided on the brand *Binti Shupavu,* meaning strong or fearless young woman in Swahili.

After determining the initial intervention design, live prototype testing took place over 8 weeks in 10 different sites with the goal of testing the feasibility of the full intervention model in a real-world setting. Adolescent girls and their influencers in the selected communities were invited to attend Binti Shupavu programming through local mobilizers who included community volunteers, health providers, and local administrators. The team gathered data on how well the intervention performed. The design team collected user feedback primarily through participant observation and interviews. Client exit interviews were conducted with adolescent girls after their participation in Binti Shupavu (n=95) across the 5 counties in October 2021. Data suggested that girls found the intervention inspirational and appealing and that it helped them understand how contraception could help them achieve their goals (Table 5). However, we also saw that many girls (n=74) were still hesitant to participate in a session with a provider. Even among those who spoke with the provider, over half expressed that they wanted counseling or “advice” only. With further probing, many girls cited needing permission from a partner or parent before accepting a method. This suggested that additional refinement was needed around the contraceptive counseling moment. Together, we used these data to formulate recommendations for improvements to Binti Shupavu (Table 6).

**TABLE 5.** Adolescent Girls' Feedback on What They Learned at Binti Shupavu

Feedback Item	No. (%) N=95
I believe using a method to prevent or avoid getting an unintended pregnancy is important to help me achieve my goals for life.	
Agree or strongly agree	94 (99)
Disagree or disagree strongly	1 (1)
What were the most useful things that you learned during this visit today?^a^	
How to delay and space pregnancies	67 (71)
Identifying my goals and that of my family	58 (62)
Other health issues	31 (33)
How to plan to achieve my goals and that of my family	29 (31)
Other	20 (22)
Self confidence	11 (12)
How to talk to my husband/partner about goals for our family	8 (9)
Menstrual health	2 (3)

a Multiple responses possible. Results total more than 100%.

**TABLE 6.** Live Prototyping Insights and Recommendations for Adaptation to Binti Shupavu in Kenya

Insight	Recommendation
Girls were interested in the Y-Facility prototype, but they expressed a desire to have these girl-friendly spaces and service delivery moments closer to their communities and wanted them to address more diverse health topics.	Test different adaptations to the Y-Facility model to reach girls in harder-to-reach areas and incorporate additional information on health topics outside of contraception that interest girls (e.g., sexually transmitted infections).
Girls wanted to see the providers on their own schedule to receive contraception, which required repeat attendance at the facility to achieve contraceptive uptake. “Let me go think about it then I will come back next week even as I come along with my friend to also attend the sessions.” —Adolescent girl, Migori	Work with providers to be more flexible to girls' preferred timing during individual counseling moments. Continue to refine and strengthen the contraceptive service delivery moment as “opt-out,” so there is the expectation that all girls should speak with the provider during the event, even if it is just to ask questions or learn more.
Despite targeting married adolescent girls and teen mothers, other cohorts of girls participated. Inconvenient timing and the need to complete other tasks affected attendance of key audiences, particularly among married adolescent girls. “I was told about the sessions but I have to go wash clothes to get paid because my child depends on me.” —Adolescent girl, Kilifi	Shift mobilization approaches so that girls can participate at a time and day that works for them. Use all available mobilization channels, including phone calls and SMS, where appropriate.
Service providers were overwhelmed with a high workload, and service sites were understaffed, resulting in limited time to attend to girls. “I am the only one who provides [family planning] services and I also attend to other patients so I will attend girl sessions if I have cleared the queue.” —Service provider, Narok	Maximize the health service providers' technical expertise but try to task shift some of the role of implementation to other cadres (such as community health volunteers) who could relieve some of the heavy burden on service providers.
Girls liked the Binti Shupavu brand but were confused that each of the 3 components had a different name.	Align all components of the intervention so it points in a consolidated way toward the Binti Shupavu brand (e.g., rename Y-Facility to Binti Shupavu Clinic, rename Mastory za Kikwetu to Binti Shupavu Stories).
Girls were interested and excited by the skills classes in advance of the community fair. However, the classes were time-consuming for the staff to plan and conduct. “From the lessons received I moved from zero to somewhere. I love coming and meeting my peers who have gone through the same thing, I've realized that [I] am not alone.” —Adolescent girl, Kilifi	Refine the skills sessions based on available resources and scale down the number of classes being provided to maintain girls' interest within the confines of available resources.
The Mastory za Kikwetu prototype doubled as a community entry point because 67% of girls were referred to the Y-Facility during live prototyping through the Mastory za Kikwetu sessions.	Initiate Binti Shupavu stories ahead of Binti Shupavu in-clinic sessions to drive community buy-in and mobilization.
Girls tended to connect better with youthful mobilizers and community health volunteers. “We prefer the skills session to be conducted by someone youthful to allow us to freely ask questions and express ourselves.” —Adolescent girl, Homabay	Build out the role of youth mentors within the intervention, using the example of Matasa Matan Arewa mentors, Kuwa Mjanja Queens, and other cadres in A360's implementation geographies.

Abbreviation: SMS, short message service.

During live prototyping, we were able to accelerate problem-solving by leveraging lessons learned in the other program geographies. As challenges or questions arose in Kenya that were encountered in the first investment phase, project staff on the global team connected the Kenya team members with key staff members from other country programs to share experiences and brainstorm solutions together. The following key topics were explored.

1. Keeping girls engaged during the facility-based sessions: Girls did not always come on time to the Y-Facility session. This resulted in attrition or frustration from girls waiting for activities to start. Kenya borrowed from a technique from the project's intervention in Tanzania that included facilitation of games during these wait periods to keep girls engaged—something that the Tanzania team tested in 2019 when it started to see girls leaving events before receiving counseling during lulls in activity.
2. Facilitating an opt-out contraceptive service delivery moment: Seeing that many girls were hesitant to speak with providers during the Y-Facility prototype for fear that it would stigmatize them, Kenya implemented an “opt-out” provider visit approach. Borrowed from Tanzania, this approach makes it standard for all girls to receive a one-on-one counseling moment. Making it a default for all girls to attend counseling destigmatizes care-seeking behaviors and gives girls privacy to discuss whatever they wish with the provider.
3. Outreach-based events: Girls were eager to have service delivery spaces closer to them. The design team pulled lessons from Tanzania's out-of-clinic community-based pop-up events and Nigeria's outreach events to explore how the Y-Facility prototype could be operated outside of the static facility setting. This allows the intervention to take services closer to the girls for whom distance is a barrier to accessing health services.
4. Peer connections: We noted that with little formal support, girls were motivated to support young women like themselves. Girls tended to connect better with youthful mobilizers and community health volunteers. Borrowing from the program's youth cadres in Tanzania (Kuwa Mjanja Queens) and in Nigeria (Big Sistas), Binti Shupavu sought models to incorporate more youthful voices in the intervention and establish tools and processes for engagement in the intervention's delivery and ongoing improvement.

### Final Intervention Design

**FIGURE 7:**
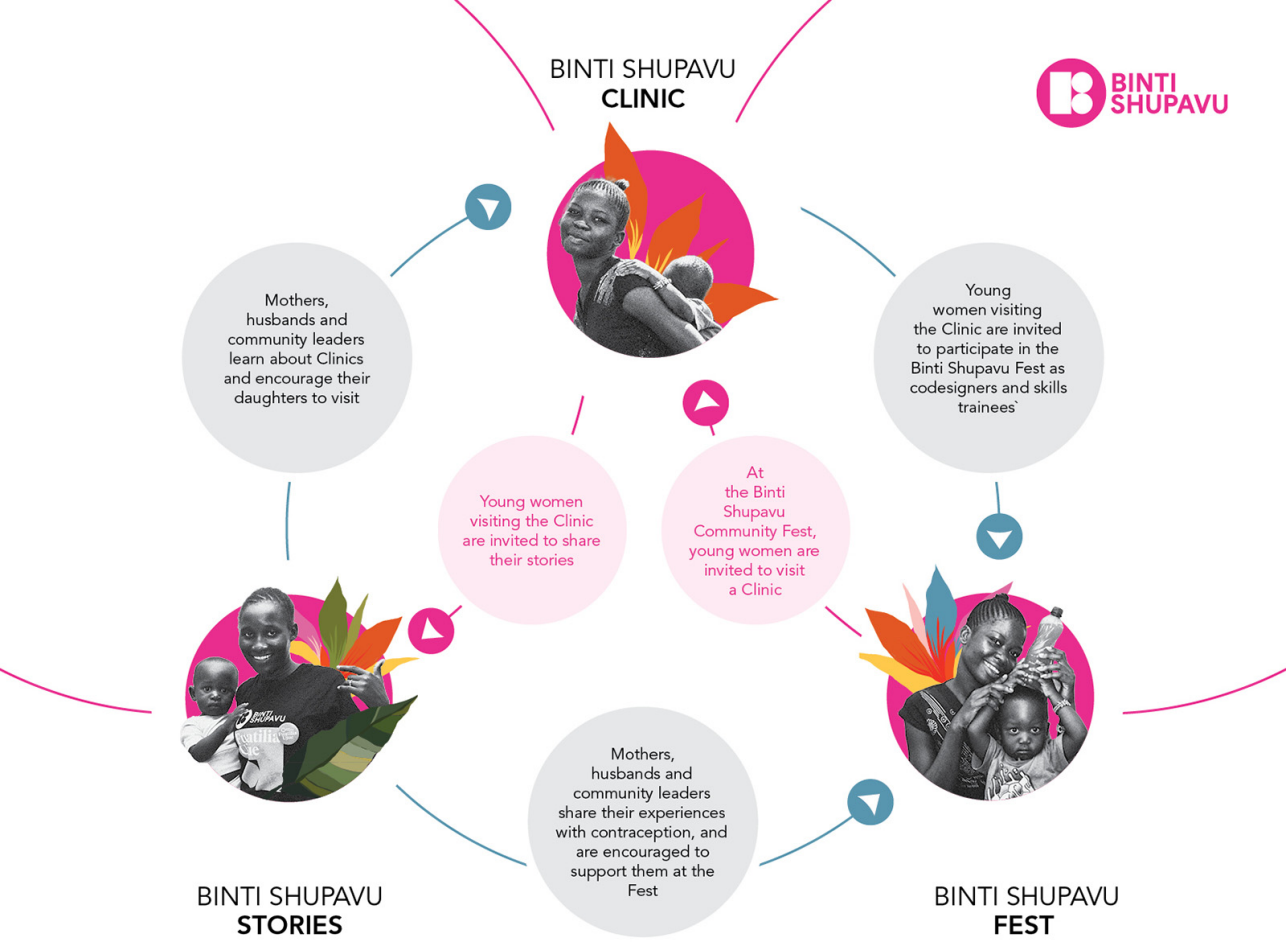
Final Binti Shupavu Intervention Design

At the end of live prototyping, the design team finalized the Binti Shupavu intervention model by incorporating the feedback received (Figure 7) and adjusting the design of components in response to this feedback. Binti Shupavu is designed to tap into girls' aspirations, understanding their experiences and placing their needs first. The intervention supports girls' agency and contraceptive decision-making by creating a safe space (Binti Shupavu Clinic) for young women to build trust in the health system, learn about contraception, and share experiences and stories with their peers. It works with providers to improve their counseling capacity to address young women's concerns and promote the provision of a full range of contraceptive options. The intervention engages and educates influencers in the community and those closest to young women (via Binti Shupavu Stories sessions) so that they can collaboratively address misinformation and support the decisions girls make about their bodies and futures. This culminates in a moment for girls and those around them to come together to celebrate the unique contributions of young women within their communities (Binti Shupavu Fest). The intervention creates further opportunities for smaller groups of adolescent girls to gain additional skills and to cocreate plans for this celebration moment together with the implementation team. This additional training gives girls the opportunity to gain a broader set of knowledge and be empowered and equipped with new skills to link them to economic opportunities. Through Binti Shupavu, young women gain accurate information and receive support to make decisions about their bodies and their futures with confidence.

Binti Shupavu is a Kenyan experience on a user journey that our global adolescent reproductive health program now understands very well. Girls feel contraception is relevant for them; they are supported in a way that feels aspirational and goes beyond health; their key influencers are engaged; and services are local, relevant, and friendly. We strove to replicate this approach in a fit-for-purpose way through contextualized stories and branding, working with the health system that exists in the geographic context, and being curious about and accommodating to the wide diversity of girls who need services locally.

### Early Implementation Results

**FIGURE 8:**
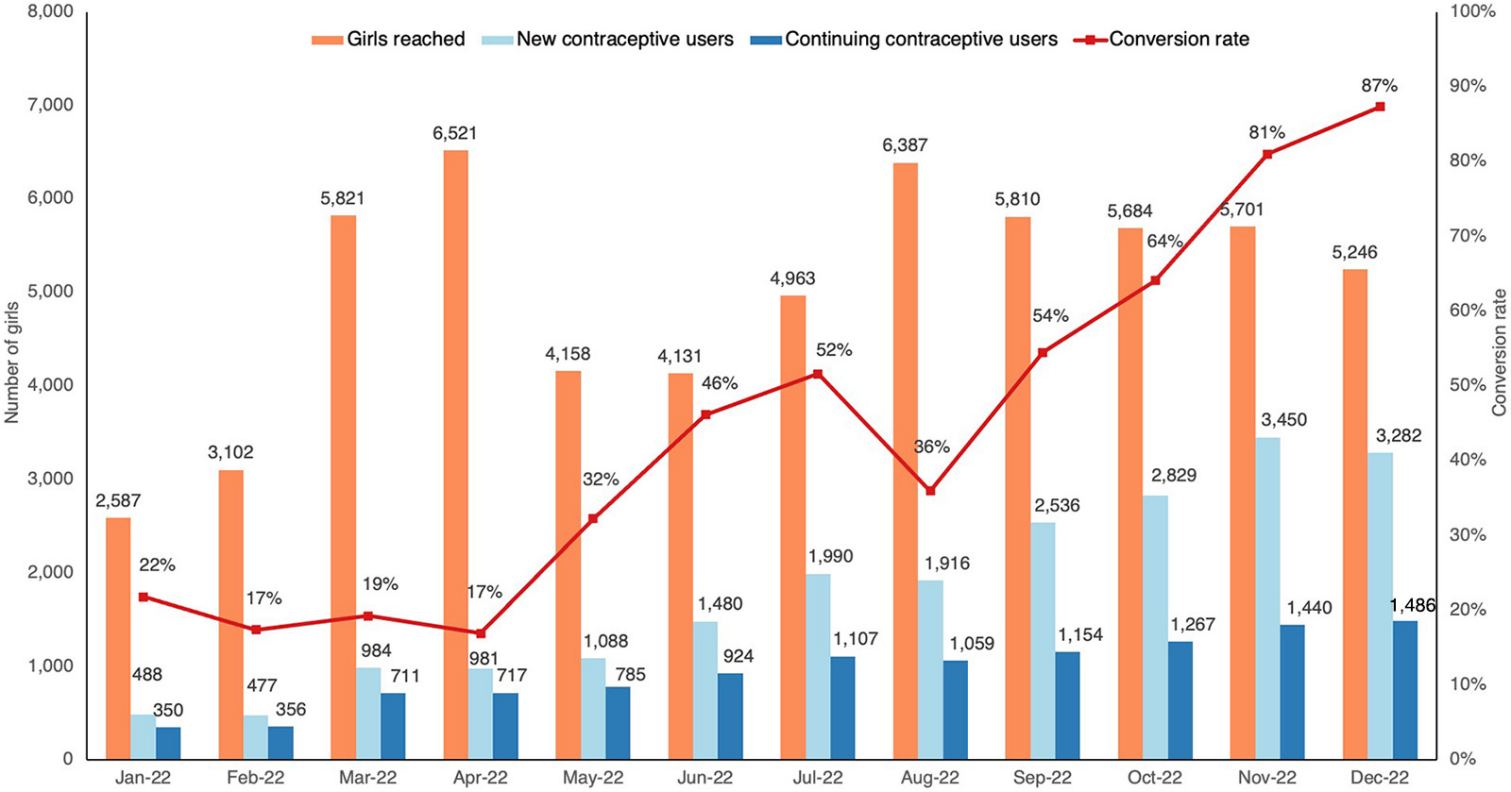
Performance of Binti Shupavu Intervention, Kenya, 2022

In the first year (January–December 2022), the intervention reached 60,111 adolescents aged 10–19 years. Of these, 21,698 were new contraceptive users (36%), while an additional 3,873 (19%) were continuing users. We scaled Binti Shupavu from 90 facilities at the end of 2021 to 360 facilities at the end of 2022. The new user reach increased every month throughout the course of the year (Figure 8). This increase is the result of more than just an increased geographic footprint: the total number of facilities increased 4-fold from January to December 2022, while new contraceptive users per month increased 7-fold. This demonstrates that the intervention also became more effective at reaching new users even as it scaled. Conversion rate, a marker of success both in targeted mobilization and in contraceptive counseling coverage, also showed significant improvements over the year, from 22% in January to 87% in December 2022.

**FIGURE 9:**
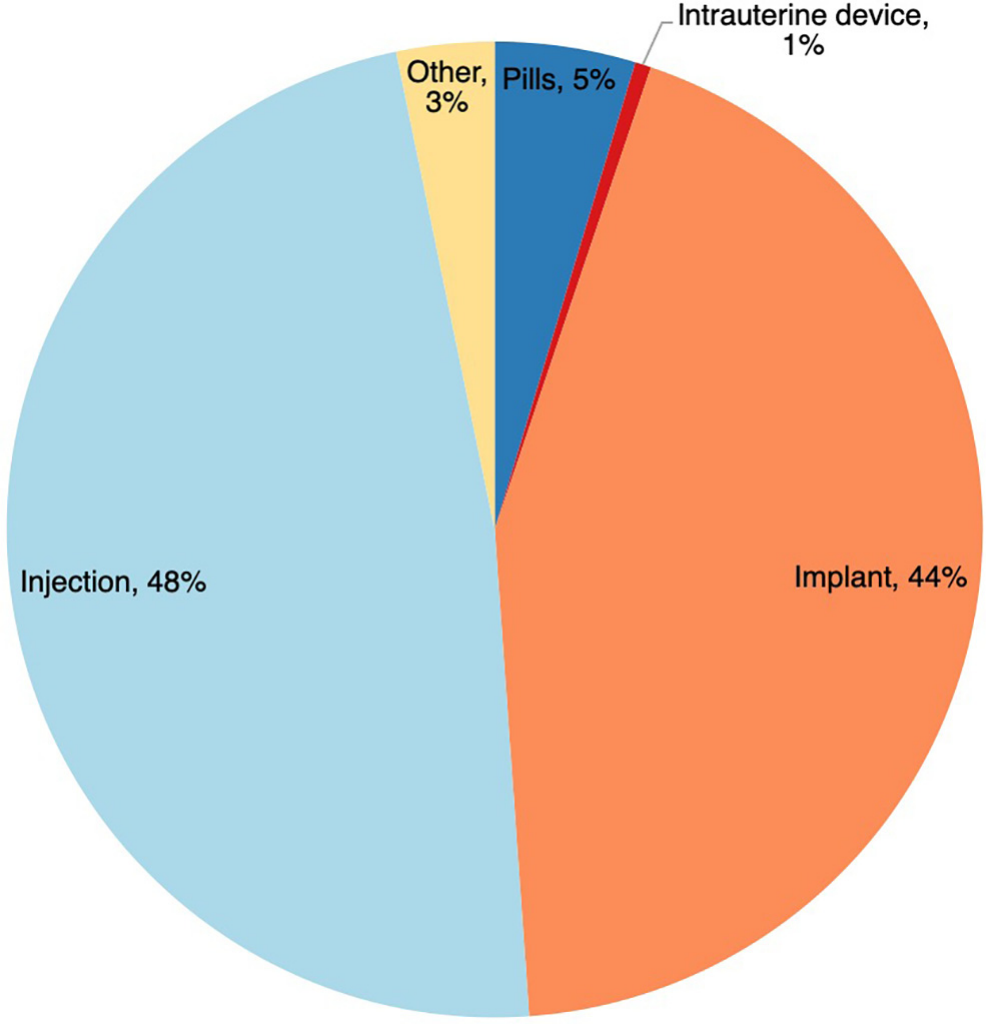
Contraceptive Methods Chosen by New Contraceptive Users Through Binti Shupavu, Kenya, 2022

Forty-eight percent of new contraceptive users supported through Binti Shupavu in 2022 selected an injectable contraceptive method, followed closely by implants (44%) (Figure 9). Given that only 37.1% of contraceptive users nationally are using an implant, high rates of implant uptake among this population are a promising indication that the intervention is removing barriers for girls to take up contraceptive methods that are aligned with their preferences.^30^

**FIGURE 10:**
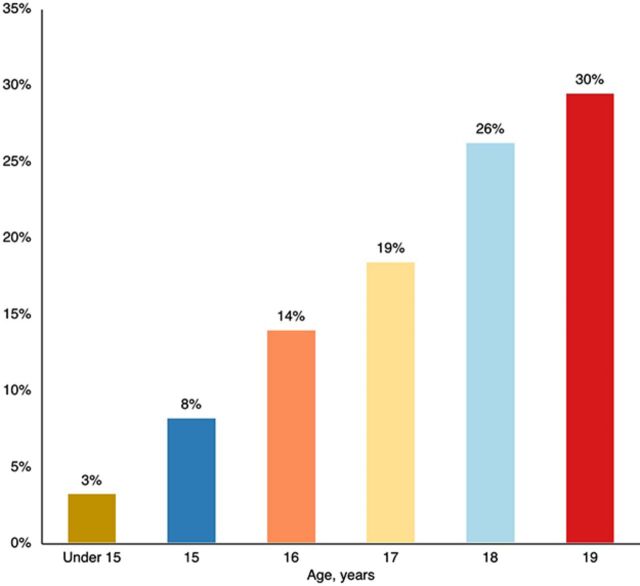
Age of New Contraceptive Users Through Binti Shupavu, Kenya, 2022

A little more than half (56%) of new contraceptive users supported through Binti Shupavu in 2022 were aged 18 or 19 years, which is to be expected because contraceptive need increases as girls get older (Figure 10). One-quarter of new contraceptive users were aged 16 years or younger, suggesting that the intervention effectively reaches younger girls who often face greater barriers to contraceptive uptake.

Differences in context, such as size of the target population, strength of the health system, and norms around family planning, make it impossible to make a perfect comparison. However, how Binti Shupavu performed in the first year against our other interventions in their initial implementation phase helps us understand if our replication efforts were successful. In Kenya, we were able to expand the intervention's reach rapidly and outperformed all other geographies except Tanzania. Kenya had the highest number and proportion of continuing users, which is likely due to the higher rates of contraceptive uptake among adolescent girls in Kenya when compared to the other geographies. Kenya and the southern Nigeria intervention, 9ja Girls, share the lowest conversion rate (44%), but as previously noted, Kenya's conversion rate improved throughout the year as mobilization became more targeted and the opt-out counseling moment during clinic sessions was strengthened. Overall, monitoring data from Binti Shupavu suggest that the intervention in Kenya can be favorably compared to the other interventions and that we were able to successfully replicate the user journey for contraceptive uptake in a Kenyan context (Table 7). The question of effectiveness of the user journey to sustain long-term contraceptive use among adolescents in Kenya will be answered through an ongoing external evaluation to be concluded by the end of 2024.

**TABLE 7.** Binti Shupavu in Kenya Compared to Other A360 Programs in Different Countries

First Year Program Monitoring Data	Reach, No.	New Users, No.	Continuing Users, No.	Conversion Rate, %	LARC Users, %
Ethiopia – Smart Start	29,146	14,833	7,245	77	23
Nigeria – 9ja Girls	38,321	16,206	1,529	44	18
Nigeria – Matasa Matan Arewa	5,834	3,308	30	57	26
Tanzania – Kuwa Mjanja	99,632	59,292	4,406	62	63
Kenya – Binti Shupavu	60,111	21,501	11,356	44	44

Abbreviation: LARC, long-acting reversible contraception.

## LESSONS LEARNED ABOUT REPLICATION USING HCD

The following reflections summarize what we see as the benefits, limitations, and opportunities for the application of HCD during a replication process and may be useful for others interested in applying HCD in a similar way.

### The Value of an HCD Replication Approach

In this case study, we argue that HCD is a promising tool for navigating the replication of evidence-based practice in a new context. The complexity of health systems and the diversity of populations they serve mean that it is not possible, nor desirable, to replicate an evidence-based intervention with perfect fidelity in a new setting. Instead, implementers must make challenging decisions about what to keep and what to adapt to maximize the chances of success. The benefits of design in public health more generally—keeping user perspectives at the center, solving problems through testing to learn, and collaborating with communities and organizations—served our replication efforts.^8^

We argue that HCD is a promising tool for navigating the replication of evidence-based practice to a new context.

The design team was able to use HCD approaches to define the target population and understand their needs at a level deeper than demographic analysis permitted. This helped guide us in identifying promising intervention components from other geographies to test. HCD mindsets of cocreation and listening to users meant that, even as the goal was replication, the design team was open to incorporating new components. Many of the sacrificial concepts that we tested during the initial phase of this Kenya design process did not appeal to adolescent girls in Kenya despite being effective components of our interventions elsewhere. In these instances, feedback from users sparked new ways of delivering messages or providing services that were refined over time with input from adolescent girls, providers, and community members. Additionally, using HCD to replicate the user journey for contraceptive uptake was a simpler, faster process than designing a new intervention. Few of our staff members had experience with design; thus, there was a learning curve to adapt to and implement HCD mindsets and approaches. However, having a blueprint for what we hoped to build made it easier to assess progress and evaluate the outcomes of prototyping. By building on what already existed and adapting based on users' needs, we were able to quickly assemble the core components of our proposed intervention. We approached the HCD replication process as fluid and continuous, which meant we were able to benefit from not just the original intervention models but also from the lessons learned through testing and implementation. The application of the full scope of existing program learning allowed us to take advantage of valuable insights that expedited the refinement of the intervention. This, in turn, accelerated our design timeline from approximately 18 months in the first phase to about 10 months for replication. We urge others who are considering using HCD to replicate an intervention to draw on previous lessons throughout the process, including the early implementation phases.

### Cautions and Considerations in the Use of HCD for Replication

#### Determining Effectiveness

As with any tool, there are limits to what design can offer. The goal of replication is to adapt evidence-based interventions to a new setting to make better use of scarce resources and improve health outcomes. However, replication efforts are often ineffective, and it can be challenging to identify why. As we have argued, HCD has potential to support the development of more desirable products, services, and interventions, and design can be completed more quickly when there is already an understanding of what conditions and prompts support users to change their behavior. In the case of A360, our approach builds on what is known to work for AYSRH demand and services—training health workers, improving facility friendliness, and generating demand through multiple channels.^31^ Yet, the layering of an additional focus on tapping into girls' aspirations and making the link to contraception as a tool for them to achieve their goals is different from many traditional approaches. We recognize that the lessons learned in HCD are no substitute for traditional public health methods used to determine the effectiveness of interventions.^32^ Although we are optimistic that our own monitoring suggests we have successfully replicated our user journey and that initial use of contraceptive methods by adolescent girls has been high, the short time frame of the design process and monitoring data are not able to show us if girls are continuing to use those methods to act on their own fertility preferences to delay and space children. Additional studies are being conducted to look at the rate of contraceptive continuation as well as assess the population-level impact of Binti Shupavu on modern contraceptive use among adolescent girls in the intervention areas. As others have called for, although we view design as an essential tool, it is evolving and should be used in collaboration with cross-disciplinary tools and resources in service of health impact.^8^

#### Resource Intensive

Even when used for replication, HCD processes are often more resource intensive than anticipated. Although the design process in Kenya was faster, at 10 months, it was still a significant use of staff time and resources. Given that we deeply understood the challenge of adolescent contraceptive use from our work in other geographies, an additional opportunity to streamline the design process includes skipping the “insight gathering” phase. In our process, we spent about 2 months preparing and executing the initial inquiry phase, and our results ultimately reflected both the evidence base and what we had seen in other program geographies. We could have begun with clearly stated assumptions about our target audience and their needs and moved directly into ideation and prototyping based on the intervention components that already existed in our other geographies. This would have allowed us to take full advantage of design's strengths in global development, such as collaboration with communities and building to learn, without spending time relearning what we already knew. Additional tools to support this targeted testing of key intervention components would be a valuable contribution to the community of practice.

#### Setting Expectations

We could have done more to align our internal teams on what to expect during an HCD replication process, and we recommend others using this approach take the time to set expectations with their own team. HCD is often misunderstood and requires work to align.^33^ This is even more true of design replication, which is not well understood in the evidence base nor by design practitioners. Among our team, we had to reinforce that borrowing ideas from what had worked before, even early in the design process, was a potentially powerful tool in a context such as ours, where much was already understood about the problem and pathways to solutions.

### HCD as a Vehicle for Localization in Replication of Evidence-Based Practice

As a result of travel limitations imposed on us because of the COVID-19 pandemic, only our Kenya-based team could conduct prototyping and observe testing in person. Although our original intent in the second phase of the project was to transfer more ownership over the HCD process to country-based leadership rather than external consultants or U.S.-based staff, the pandemic accelerated the speed at which this happened and the extent to which it was required. Localization of decision-making and building capacity of more diverse teams to conduct HCD aligns with the spirit of design and the goal of developing more equitable, sustainable solutions.

A challenge that arose for us was the need to support local decision-makers to be aware of and use the evidence and implementation experience from earlier work. The design team worked hard to minimize the feeling of distance by using online tools and sharing pictures and voice memos, but it was not possible to convey everything. This poses a particular challenge for HCD replication processes. One of the benefits of replication is that project implementers are already familiar with the early signs of behavior change or problems. Although we did our best to communicate these nuances virtually, the HCD process is one that floods teams with data. It is difficult to synthesize that data rapidly and identify what intervention components will be the most important to achieving the project's goal. An experienced implementer can help teams prioritize which iterations to address first and should be an important resource when replicating.

A challenge that arose for us was the need to support local decision-makers to be aware of and use the evidence and implementation experience from earlier work.

For example, we knew from our previous experience that properly targeted mobilization, or the recruitment of eligible young women to attend program activities, supports program efficiency by ensuring services reach girls in need. Following the design process, improving mobilization was among several recommendations made and took several months to achieve. We speculate that we could have improved contraceptive uptake more quickly if a staff person from Tanzania or Nigeria had been able to come in person, as our other geographies also struggled with this element initially. This suggests there is a vital role for national, regional, and global experts to play in service of more effective localization, and we encourage other practitioners who are considering applying this approach to support local decision-making and make use of in-country support wherever feasible.

## Notes

**Peer Reviewed**

**First published online:** December 15, 2023.
